# The Effect of Implementation of Guideline Order Bundles Into a General Admission Order Set on Clinical Practice Guideline Adoption: Quasi-Experimental Study

**DOI:** 10.2196/42736

**Published:** 2023-03-21

**Authors:** Justine Mrosak, Swaminathan Kandaswamy, Claire Stokes, David Roth, Jenna Gorbatkin, Ishaan Dave, Scott Gillespie, Evan Orenstein

**Affiliations:** 1 Hennepin Healthcare Minneapolis, MN United States; 2 Department of Pediatrics Emory University School of Medicine Atlanta, GA United States; 3 Division of Pediatric Hematology/Oncology Department of Pediatrics Emory University School of Medicine Atlanta, GA United States; 4 Children's Healthcare of Atlanta Atlanta, GA United States; 5 Department of Pediatrics University of Pittsburgh School of Medicine Pittsburgh, PA United States

**Keywords:** clinical practice guideline, user-centered design, clinical decision support, diagnostic uncertainty, diagnostic, decision support, CPG, clinical guideline, order bundle

## Abstract

**Background:**

Clinical practice guidelines (CPGs) and associated order sets can help standardize patient care and lead to higher-value patient care. However, difficult access and poor usability of these order sets can result in lower use rates and reduce the CPGs’ impact on clinical outcomes. At our institution, we identified multiple CPGs for general pediatrics admissions where the appropriate order set was used in <50% of eligible encounters, leading to decreased adoption of CPG recommendations.

**Objective:**

We aimed to determine how integrating disease-specific order groups into a common general admission order set influences adoption of CPG-specific order bundles for patients meeting CPG inclusion criteria admitted to the general pediatrics service.

**Methods:**

We integrated order bundles for asthma, heavy menstrual bleeding, musculoskeletal infection, migraine, and pneumonia into a common general pediatrics order set. We compared pre- and postimplementation order bundle use rates for eligible encounters at both an intervention and nonintervention site for integrated CPGs. We also assessed order bundle adoption for nonintegrated CPGs, including bronchiolitis, acute gastroenteritis, and croup. In a post hoc analysis of encounters without order bundle use, we compared the pre- and postintervention frequency of diagnostic uncertainty at the time of admission.

**Results:**

CPG order bundle use rates for incorporated CPGs increased by +9.8% (from 629/856, 73.5% to 405/486, 83.3%) at the intervention site and by +5.1% (896/1351, 66.3% to 509/713, 71.4%) at the nonintervention site. Order bundle adoption for nonintegrated CPGs decreased from 84% (536/638) to 68.5% (148/216), driven primarily by decreases in bronchiolitis order bundle adoption in the setting of the COVID-19 pandemic. Diagnostic uncertainty was more common in admissions without CPG order bundle use after implementation (28/227, 12.3% vs 19/81, 23.4%).

**Conclusions:**

The integration of CPG-specific order bundles into a general admission order set improved overall CPG adoption. However, integrating only some CPGs may reduce adoption of order bundles for excluded CPGs. Diagnostic uncertainty at the time of admission is likely an underrecognized barrier to guideline adherence that is not addressed by an integrated admission order set.

## Introduction

### Problem Description

Clinical practice guidelines (CPGs) are designed to help standardize and disseminate evidence-based practices for various disease processes. Implementation of CPGs has been shown to decrease variation in care delivery, reduce costs, and improve patient outcomes [1-3]. However, clinician adherence to CPGs in many contexts remains suboptimal, impeding the delivery of high-value patient care [4,5].

Clinical decision support (CDS) systems integrated into the electronic health record can address some of these barriers and improve CPG adoption [6]. For example, order bundles serve as the building blocks for comprehensive order sets, which allow physicians to place multiple evidence-based orders for a single diagnosis with a few keystrokes without having to search individually for each order. CPG-associated order sets aggregate CPG-recommended therapies into a single order set, reducing the cognitive and physical work burden on clinicians to follow guidelines [6-9]. The use of CPG-associated order sets has improved outcomes in sepsis, pneumonia, and many other diseases [6,7,10,11].

Despite this evidence, we found that many CPG-associated order sets were used in <50% of eligible encounters in our own health system. The lack of order set adoption leads to less penetration of CPGs into clinical practice and reduces CPG impact on outcomes.

### Available Knowledge

Research into guideline nonadherence thus far has demonstrated that barriers to guideline adoption are often context-specific and difficult to generalize to different settings [5]. In a systematic review of clinician surveys investigating potential barriers to guideline adherence, commonly identified barriers included lack of awareness or familiarity, lack of agreement or outcome expectancy, inertia from previous practice, and existing external barriers [5].

Order bundles embedded into larger order sets can be a powerful tool in improving CPG adoption [6,12]. For example, Munasinghe et al [10] incorporated multiple CPG order bundles into admission order sets and demonstrated improved adoption. However, unintended consequences, including increased physical and cognitive workload, can also result when these order sets demonstrate poor usability or are implemented at the wrong time in the clinician workflow [4,8]. Insufficient customization of order set content, mismatches between technology and human practices, and inadequate maintenance and modification of order sets have all been shown to contribute to order set nonuse and subsequent exposure to potential medical errors [4,8,9].

Furthermore, diagnostic uncertainty and increasing patient complexity also contribute to guideline nonadherence, which may be appropriate in certain contexts [13-15]. Single-diagnosis guidelines may be too simplistic to apply to the majority of patients [16]. Diagnosis codes have proven to be a poor marker of these barriers [14,15,17], and a lack of clear definitions continues to make them difficult to measure [14].

It remains unknown what CDS designs best address these barriers and most improve CPG adherence.

### Rationale/Specific Aims

Our purpose for this project was to provide higher-value care through improved adherence to evidence-based CPGs at our institution. In preliminary data described elsewhere [18], we found that common reasons among local frontline providers for not adopting CPG order sets in eligible populations included lack of awareness (32%) and forgetting to use the stand-alone CPG-specific order set (20%). We therefore implemented a new CDS system in our admission process in the form of embedded CPG order bundles integrated into the general pediatrics admission order set that were identical to CPG-specific order bundles in the existing stand-alone CPG-associated order sets. Our primary aim was to increase CPG-specific order bundle use for eligible patients admitted on the general pediatrics service by 20% from July 2019 to May 2021. The primary aim was determined by the project stakeholder team to likely be an achievable improvement based on initial data that many CPGs demonstrated an <50% adherence rate, as well as an improvement that would justify the anticipated effort to complete the project. Secondary aims included determining if there were differences in order bundle use between specific CPGs at the intervention site and comparing CPG order bundle adherence between the intervention site and another hospital within the same health system where the intervention was not implemented.

## Methods

### Context/Setting

This study was performed on the general pediatrics service in an academic urban children’s hospital within a 3-hospital, 638-bed pediatric health system serving the greater Atlanta, Georgia, area. Over 90 pediatric and family medicine residents rotate through the general pediatrics service each year and are overseen by 16 pediatric hospital medicine faculty members at the intervention hospital. Our institution uses an official electronic health record supplied by Epic Systems. Currently, there are over 20 general pediatric-specific CPGs customized to local workflows that are available for use through the institutional intranet. Prior to the intervention, 15 of the CPGs had their own stand-alone order set to facilitate adherence.

The intervention was implemented at 1 of 3 freestanding children’s hospitals; this was an academic tertiary care center staffed primarily by resident teams with pediatric hospital medicine attendings. The nonintervention site was a hybrid community-academic hospital primarily staffed directly by pediatric hospital medicine attendings within the same health system.

### Intervention

#### Planning the Intervention

Stakeholders included representatives from the Pediatric Hospital Medicine service, the Department of Clinical Effectiveness, and the Department of Quality and Safety, as well as a clinical informaticist, a human factors engineer, a medical student, and a quality improvement methodology expert. This team met formally multiple times in the planning stages of the project and while formal problem analysis was underway before the intervention.

Preintervention problem analysis has been previously described [18]. Briefly, we identified patients eligible for a CPG order set for whom it was not ordered; we contacted the admitting provider within 2 weeks, inquired about reasons for CPG nonuse from a predefined list (we also added categories as needed), and asked for narrative comments. Based on these results, we created a Pareto chart that identified the most common barriers to CPG order set use: (1) lack of awareness or forgetting to use the CPG (2), eligibility for multiple CPG order sets at the time of admission, and (3) use of a similarly named order set that was not the intended CPG order set.

#### The Intervention

**Figure 1:**
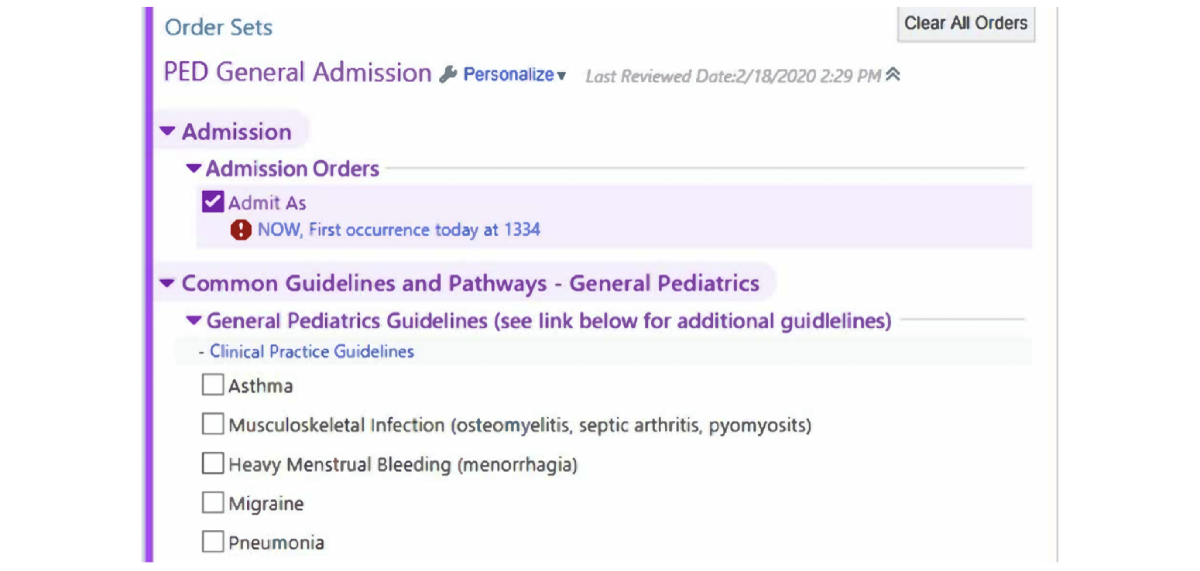
Integrated general pediatrics admission order set with clinical practice guideline order bundles.

CPG-specific order bundles were integrated into the general pediatrics admission order set for better visibility and more efficient usability. Order bundles for 6 CPGs were chosen for the intervention, including asthma, complicated pneumonia, heavy menstrual bleeding, migraine, musculoskeletal infection, and uncomplicated pneumonia. These incorporated CPGs were chosen because they either (1) demonstrate low guideline order set use, (2) are very common, or (3) represent important improvement areas in antimicrobial stewardship. Order bundles were added to a section titled “Common Guidelines and Pathways—General Pediatrics” (Figure 1).

Orders in each CPG order bundle were identical to the existing stand-alone CPG-associated order sets. Within each order bundle, embedded hyperlinks referenced the published CPG and relevant literature from which recommendations were made and referenced common target disease pathogens for bundles that recommended empiric first-line antibiotics. For patients that qualified for multiple CPG order sets, the integrated order set also allowed for the selection of multiple relevant order bundles within the order set.

Prior to implementation of the integrated order set into a live production environment, formative usability testing and summative usability testing were both completed. Usability testing aimed to test the effectiveness and iteratively improve the intervention in a simulated environment. Results are reported elsewhere [18].

To address the barrier of similarly named but non-CPG order sets being used accidentally, we identified order set “mimics” by searching the system with common clinical synonyms for each CPG. Similarly named order sets were retired from the production environment after obtaining approval from both the order set owners and the corresponding CPG owners. In total, 9 mimics were identified, and all of these were subsequently retired after owner approval. Additionally, all relevant CPG-associated order sets were reviewed and updated, if necessary, for both naming consistency and related search terms.

After the integrated order set was implemented into a live production environment, an update outlining the new CDS tool and its capabilities and expected use was emailed to all current and incoming residents to review and presented at the weekly resident educational conference.

### Study of the Intervention and Measures

All patients aged 0 to 21 years who were admitted to the general pediatrics service and met the eligibility criteria for any one of the incorporated CPG order sets based on preexisting computable population definitions were included in this study. This study used a quasi-experimental design, analyzing pre- and postintervention CPG order set adherence at both the intervention site and a nonintervention site. Our primary exposure was intervention period, with the preintervention period defined as July 1, 2019, to June 3, 2020, and the postintervention period as June 4, 2020, to May 28, 2021, as the integrated order set went live on June 4, 2020, at the intervention site.

Our primary outcome was the proportion of appropriate CPG order set use for eligible patients at the time of admission. To evaluate the impact of our intervention, we adopted existing automated queries to assess whether the clinician used the appropriate CPG order set, a wrong but similarly named order set, or the available general pediatrics admission order set. We also reviewed whether the “CPG guideline initiation order” was signed, which is a prechecked order in all our included CPG order sets. Through the query, demonstrated use of the CPG-associated order bundle and the presence of the guideline initiation order were assumed to represent appropriate guideline order set use. Encounters where the clinician appeared to use the appropriate guideline order set or bundle but the guideline initiation order was absent were manually chart reviewed to confirm appropriate order set use. All encounters that appeared eligible but where the clinician did not appear to demonstrate appropriate guideline order set use were manually chart reviewed to ensure CPG eligibility at admission throughout the study period. Eligibility was based on defined eligibility criteria in each published CPG. All manual chart review was completed by a pediatric hospital medicine fellow using both the Epic electronic health record and the Phrase Health system.

The pre- and postintervention proportion of CPG-eligible admissions for which the CPG order set or bundle were used was compared at the intervention hospital, where the integrated order set was implemented into the production environment. Data in this context were considered our “intervention cohort.” The proportion of appropriate CPG order set use for eligible patients was also compared in the same study period at the nonintervention hospital within the same health system, where the integrated order set was not implemented. This hospital uses the same CPGs and associated order sets and serves a similar patient population in the greater Atlanta area; it was thus considered our “nonintervention cohort.” The purpose of having both intervention and nonintervention cohorts in this study was to better assess whether the observed outcomes were directly related to our intervention rather than secular trends.

In this intervention, there was concern that surfacing some guidelines in the integrated order set but not others could lead to the unintended consequence of reducing order set use of CPGs that were not included in the intervention. Therefore, pre- and postintervention use of CPG order sets that were not initially included in the integrated admission order set (acute gastroenteritis, croup, bronchiolitis) was assessed as a “balancing cohort.”

### Evaluation of Diagnostic Uncertainty

As our intervention was created to address lack of knowledge and awareness of guidelines, we hypothesized that it would not address diagnostic uncertainty, an underrecognized barrier that may influence order set adoption. In a post hoc analysis, we therefore aimed to evaluate the presence of diagnostic uncertainty at the time of admission to determine if this barrier accounted for a larger proportion of CPG order bundle nonuse after the intervention.

All eligible encounters where the associated CPG order set was not used were manually chart reviewed to assess the presence of diagnostic uncertainty throughout the entire study period. Diagnostic uncertainty was defined based on an algorithm (Multimedia Appendix 1) adapted from the approach of Bhise et al [15] to measuring diagnostic uncertainty in primary care. In the algorithm, encounters needed to include direct or indirect markers of uncertainty in documentation, initial definitive treatment had to have been withheld while awaiting further diagnostic workup or observation, and an operational definition of diagnostic uncertainty had to be met. Two members of the research team, a pediatric hospital medicine fellow and a pediatric resident, completed the manual chart review based on information available in the initial history and physical documentation and reported the presence or absence of diagnostic uncertainty. Interrater reliability was assessed to confirm reliability between the 2 researchers’ assessments. After chart review, the number of eligible encounters where the CPG-associated order set was not used that demonstrated the presence of diagnostic uncertainty was compared before and after implementation to determine the change in proportion after the intervention.

### Analysis

Data were summarized using counts and percentages by site (intervention and nonintervention), period (pre- and postintervention), and guideline (eg, asthma or heavy menstrual bleeding). Binary logistic regression was used to analyze overall and by-guideline associations between use of appropriate CPG-specific order bundle (yes vs no) and period (pre- and postintervention) across sites via statistical interactions. We further ran binary logistic regression models evaluating the association between use of appropriate CPG-specific order bundle and period in the balancing cohort and relevant guidelines. Results are presented as contingency tables with odds ratios (ORs), 95% CIs, and corresponding *P* values. All analyses were conducted using SAS (version 9.4; SAS Institute), and significance was assessed at the .05 level. Percent adherence for eligible encounters by month for the intervention cohort was tracked and plotted on a statistical process *P* chart with annotations for the order set clean up and integrated order set go-live interventions.

### Ethical Considerations

This study was deemed by the Children’s Healthcare of Atlanta Institutional Review Board to be nonhuman-subjects research as a quality improvement study (STUDY00000367).

## Results

**Figure 2:**
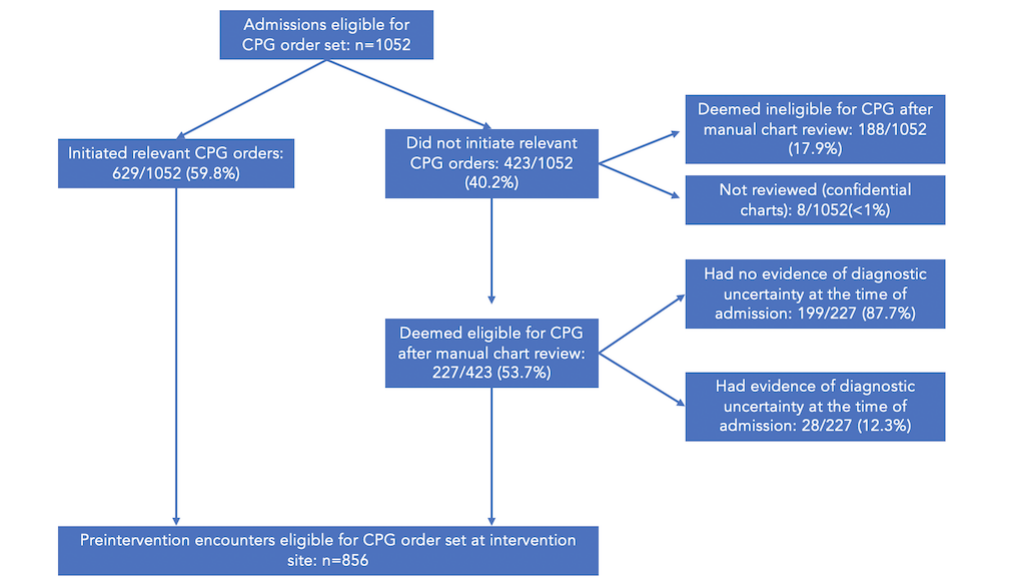
Preintervention admission encounters for the intervention cohort. CPG: clinical practice guideline.

**Figure 3:**
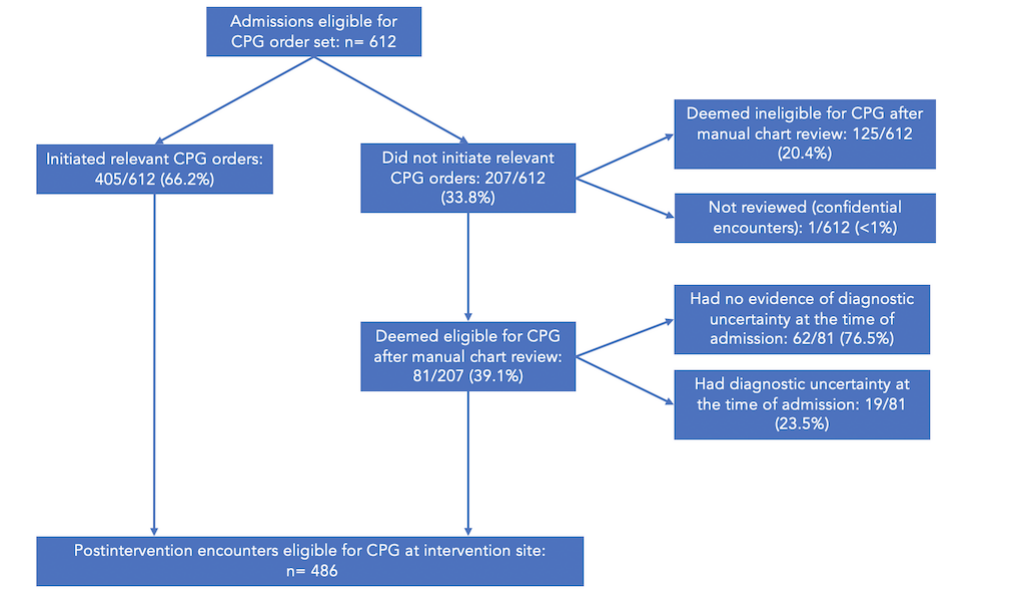
Postintervention admission encounters for the intervention cohort. CPG: clinical practice guideline.

The integrated order set went live on June 4, 2020. From January 1, 2019, to May 28, 2021, a total of 1664 encounters were identified as eligible for a CPG order set based on preexisting computable population definitions. Of these encounters, 1052 were preimplementation (Figure 2) and 612 were postimplementation (Figure 3).

The number of encounters was unbalanced, partially due to lower-than-average admission volumes as a result of the COVID-19 pandemic. We manually reviewed all encounters that appeared eligible by computable definitions for a CPG order bundle where the CPG order bundle was not used: 423/1052 (40.2%) encounters before the intervention and 207/612 (33.8%) after the intervention. Of the encounters that were reviewed, 188/1052 (17.9%) before the intervention and 125/612 (20.4%) after the intervention were excluded by manual review for not meeting eligibility criteria. Overall rates of exclusion were similar when comparing the difference before and after the intervention (17.9% before and 20.4% after the intervention; 95% CI –6.62% to 1.51%, *P*=.22).

**Figure 4:**
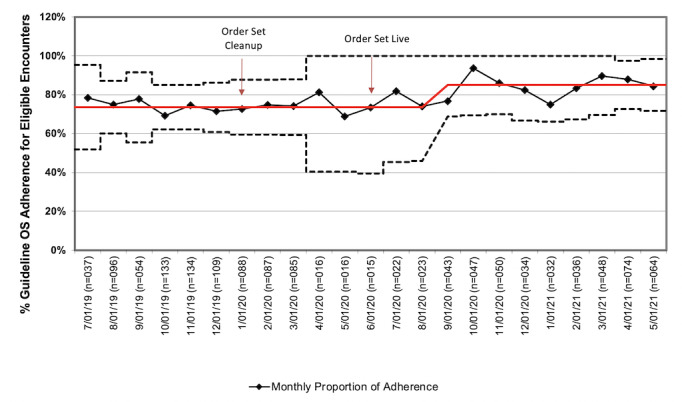
Statistical process control chart of percentage guideline order set adherence for eligible encounters at the intervention site between July 2019 to December 2020. OS: order set.

CPG order set use rates for included CPGs were tracked over time (Figure 4).

The trend in monthly adherence was positive following implementation and demonstrated special cause variation beginning in August 2020, 8 weeks after the integrated order set went live. The rate of order set use at the intervention site for integrated CPGs increased from 73.5% before the intervention to 83.3% after the intervention (OR 1.80, 95% CI 1.36-2.39). Order set use rate at the nonintervention site, where the integrated order set was not implemented but mimics were also deleted, increased from 66.3% to 71.4% during the same study period (OR 1.27, 95% CI 1.04-1.54). Of note, this increase in the nonintervention cohort appeared driven by musculoskeletal infection (OR 2.84, 95% CI 1.49-5.40) and asthma (OR 2.15, 95% CI 1.22-3.79), as seen in Table 1. When comparing ORs between the intervention and nonintervention cohorts, the intervention cohort had significantly improved order set use from before to after the intervention relative to the nonintervention cohort (intervention OR 1.80 (95% CI 1.36-2.39) vs nonintervention OR 1.27 (95% CI 1.04-1.54; *P*=.045).

When broken down by disease-specific CPGs, all integrated CPGs showed positive adherence trends after implementation in the intervention cohort but with different effect sizes. Heavy menstrual bleeding and pneumonia had more improvement than musculoskeletal infection or migraine (Table 1). Adherence in asthma, for which the CPG order set has historically high use rates, remained excellent after the intervention (92.1%-95.5%; OR 1.81, 95% CI 0.95-3.45). Adoption of CPG order bundles that were not included in the integrated admission order set (including bronchiolitis, acute gastroenteritis, and croup) decreased from 84% to 68.5% following the intervention (OR 0.41, 95% CI 0.29-0.59). Of note, this was largely driven by bronchiolitis, where adoption changed from 86.9% to 75.7% after the intervention (OR 0.47, 95% CI 0.28-0.78), as seen in Table 2.

In a post hoc analysis, based on the observation that improvements were lower for musculoskeletal infection and migraine, we reviewed 308 eligible encounters where a CPG order bundle was not used to evaluate the presence of diagnostic uncertainty at admission. One reviewer (a pediatric hospital medicine fellow) completed manual chart review on all charts and a second (a pediatric resident) reviewed a random subsample of 50 encounters (16%), with interrater reliability measured by the Cohen κ (κ=0.73, *P*<.001). The proportion of eligible encounters where the CPG order set was not used that demonstrated diagnostic uncertainty increased from 12.3% (28/227) before implementation to 23.4% (19/81) after implementation (OR 2.18, 95% CI 1.12-4.16).

**Table 1:** Order set bundle use before and after implementation on June 4, 2020, in the intervention and nonintervention cohorts.

			Intervention cohort										Nonintervention cohort													Interaction *P* value
			No bundle, n (%)		Bundle, n (%)		OR^a^ (95% CI)		*P* value			No bundle, n (%)			Bundle, n (%)			OR (95% CI)			*P* value					
**Overall^b^**										<.001												.02			.04	
	Before	227 (26.5)		629 (73.5)		Reference					455 (33.7)			896 (66.3)			Reference									
	After	81 (16.7)		405 (83.3)		1.80 (1.36-2.39)					204 (28.6)			509 (71.4)			1.27 (1.04-1.54)									
**Asthma^c^**										.07												.01			.69	
	Before	39 (7.9)		454 (92.1)		Reference					63 (10.6)			529 (89.4)			Reference									
	After	13 (4.5)		274 (95.5)		1.81 (0.95-3.45)					16 (5.2)			289 (94.8)			2.15 (1.22-3.79)									
**Heavy menstrual bleeding^d^**										.01												.24			.01	
	Before	8 (42.1)		11 (57.9)		Reference					2 (6.7)			28 (93.3)			Reference									
	After	5 (11.6)		38 (88.4)		5.53 (1.50-20.35)					8 (16.0)			42 (84.0)			0.38 (0.07-1.90)									
**Musculoskeletal infection^e^**										.30												.001			.48	
	Before	22 (75.9)		7 (24.1)		Reference					55 (67.1)			27 (32.9)			Reference									
	After	21 (63.6)		12 (36.4)		1.80 (0.59-5.44)					33 (41.8)			46 (58.2)			2.84 (1.49-5.40)									
**Migraine^f^**										.77												.08			.26	
	Before	25 (30.1)		58 (69.9)		Reference					75 (37.1)			127 (62.9)			Reference									
	After	19 (27.9)		49 (72.1)		1.11 (0.55-2.26)					81 (46.0)			95 (54.0)			0.69 (0.46-1.05)									
**Complicated pneumonia^g^**										.27												.19			.09	
	Before	7 (87.5)		1 (12.5)		Reference					28 (65.1)			15 (34.9)			Reference									
	After	5 (62.5)		3 (37.5)		4.20 (0.33-53.11)					8 (88.9)			1 (11.1)			0.23 (0.03-2.05)									
**Uncomplicated pneumonia^h^**										.03												.48			.03	
	Before	126 (56.3)		98 (43.7)		Reference					232 (57.7)			170 (42.3)			Reference									
	After	18 (38.3)		29 (61.7)		2.07 (1.09-3.95)					58 (61.7)			36 (38.3)			0.85 (0.53-1.34)									

^a^OR: odds ratio.

^b^Intervention cohort: n=1342; nonintervention cohort: n=2064.

^c^Intervention cohort: n=780; nonintervention cohort: n=897.

^d^Intervention cohort: n=62; nonintervention cohort: n=80.

^e^Intervention cohort: n=62; nonintervention cohort: n=161.

^f^Intervention cohort: n=151; nonintervention cohort: n=378.

^g^Intervention cohort: n=16; nonintervention cohort: n=52.

^h^Intervention cohort: n=271; nonintervention cohort: n=496.

**Table 2:** Pre- and postimplementation (before and on or after June 4, 2020, respectively) order set bundle use in the balancing cohort.

		No bundle, n (%)	Bundle, n (%)	Odds ratio (95% CI)	*P* value
**Overall (n=854)**					
	Preimplementation	102 (16)	536 (84)	Reference	
	Postimplementation	68 (31.5)	148 (68.5)	0.41 (0.29-0.59)	<.001
**Bronchiolitis (n=563)**					
	Preimplementation	59 (13.1)	393 (86.9)	Reference	
	Postimplementation	27 (24.3)	84 (75.7)	0.47 (0.28-0.78)	.004
**Acute gastroenteritis (n=109)**					
	Preimplementation	33 (63.5)	19 (36.5)	Reference	
	Postimplementation	35 (61.4)	22 (38.6)	1.09 (0.50-2.37)	.83
**Croup (n=184)**					
	Preimplementation	10 (7.5)	124 (92.5)	Reference	
	Postimplementation	6 (12.5)	42 (87.5)	0.57 (0.19-1.65)	.29

## Discussion

### Summary

The integration of CPG order bundles into a general pediatric admission order set improved CPG adoption in a stand-alone academic pediatric hospital compared to a control hospital within the same health system. In a post hoc analysis, the disease processes with lower diagnostic uncertainty at the time of admission saw the greatest improvement from this intervention.

### Interpretation

CPG adoption improved both in relation to preintervention encounters at the same hospital and in relation to encounters at a similar hospital within the same institution where the integrated order set was not released. This suggests that the increase in CPG adherence was directly related to the implementation of the integrated order set at the study site. While CPG adherence also significantly improved at the nonintervention hospital, the improvement seen at the intervention site was significantly more than that at the nonintervention site. Additionally, improvement was only seen for 2 of the 6 guidelines (asthma and musculoskeletal infection) at the nonintervention site, compared to all 6 guidelines showing a trend toward improvement at the intervention site. This change may reflect the removal of known CPG order set “mimics” at both locations prior to the integrated order set implementation, as this was identified as a barrier to CPG adherence in prior work. While some CPGs demonstrated improvements of close to 20%, overall improvement did not meet our initial primary aim of 20% increased adherence after the intervention. This is likely due to finding a higher than anticipated preintervention overall adherence rate, largely driven by the CPG for asthma, which has historically high adherence rates.

Nonincorporated CPGs demonstrated a reduction in order set use following implementation of the integrated order set. This finding was largely attributable to a decrease in bronchiolitis guideline adherence. The timing of this intervention, in June 2020, correlated to multiple surges of SARS-CoV-2 infections. We are unable to distinguish whether the reduction in adherence was due to our intervention or the change in the management of respiratory infections during this time. Future studies that incorporate a more comprehensive list of CPGs may elucidate how this decision support design affects nonincorporated CPGs.

The presence of diagnostic uncertainty was not initially identified as a primary barrier to guideline adherence based on frontline clinician queries during this study [18]. In our analysis, the proportion of eligible encounters without CPG adherence that demonstrated diagnostic uncertainty increased following the implementation of the integrated order set. Our intervention addressed other drivers, but not diagnostic uncertainty, which may explain a higher fraction of diagnostic uncertainty in encounters without a CPG order bundle after implementation. Alternative designs that account for the change in diagnostic certainty across a hospitalization may demonstrate a better job of improving CPG adherence.

While previous literature has shown that CPGs and associated order sets can successfully decrease variation in care delivery and improve patient outcomes [19-22], the context in which decision support is aligned into the workflow remains of utmost importance for the success of these interventions [4]. Combining alerts with order sets has been shown to have success in specific contexts [23,24] but risks generating alert fatigue and requires considerable disease-specific logic behind the alert. Alternatively, automating order suggestions through machine learned patterns of order use was shown to influence ordering behavior [4,25] but may not reflect evidence-based recommendations and can be resource intensive.

Furthermore, increasing patient medical complexity and diagnostic uncertainty leads to a workflow mismatch when coupled with single diagnoses or simplified guidelines. This gap was largely underrecognized by clinicians when self-reporting barriers to guideline adherence [18] and likely requires decision support in a different context or format to overcome than admission order sets. Mehta et al [16] sought to integrate CDS into documentation workflows through problem-oriented templates aimed at improving documentation for patients with multiple problems and to provide greater evidence-based prompts and organization. While demonstrating potential to provide recommendations during the documentation process, potentially a better context to address diagnostic uncertainty, clinicians are still called upon to label diagnoses for their patients at a point in time when this may still be unclear. Further research into the most effective format and context for CDS to address diagnostic uncertainty is needed.

### Limitations

This study has several limitations. First, results may not be generalizable, as this was a multisite, single-system study focused on a single service. Different contexts, organizational cultures, and electronic health record vendors could affect the feasibility and impact of this intervention. Second, due to the COVID-19 pandemic, hospital admission volumes were significantly lower, and admissions consisted of fewer respiratory illnesses in the postimplementation period, potentially creating pre- and postintervention cohorts that were less similar. Additionally, due to time and resource constraints, a single chart reviewer was used for charts flagged as nonadherent to confirm eligibility. Despite this limitation, pre- and postimplementation rates of exclusion were similar, suggesting this did not have a significant influence on results. Lastly, in a post hoc analysis of diagnostic uncertainty, only one reviewer reviewed all nonadherent charts to determine the presence of uncertainty. The development of an algorithm for uncertainty and an assessment of interrater reliability for a subset of charts attempted to address this limitation and minimize subjectivity.

### Conclusion

The integration of CPG-specific order bundles into a general pediatrics admission order set improved overall CPG adoption by addressing the most commonly reported barriers to CPG adherence by clinicians. Further improvement in guideline adherence could be seen with integration of a more comprehensive list of available guidelines for a particular service. Diagnostic uncertainty at the time of admission is likely an underrecognized barrier to guideline adherence that is not fully addressed with an integrated admission order set. Further work is needed to determine the impact of an integrated admission order set on clinical outcomes and what types of clinical decision support could better address the presence of diagnostic uncertainty.

## Data Availability

Deidentified data underlying this article will be shared on reasonable request to the corresponding author.

## Appendix

Multimedia Appendix 1 Overview of algorithm to evaluate for diagnostic uncertainty with case examples.

## Abbreviations

CDS: clinical decision support
CPG: clinical practice guidelines
OR: odds ratio
