# Supplementary material for: The Effect of Implementation of Guideline Order Bundles Into a General Admission Order Set on Clinical Practice Guideline Adoption: Quasi-Experimental Study
Source: JMIR Med Inform. 2023 Mar 21;11:e42736. doi: 10.2196/42736 (PMC10131941; doi:10.2196/42736)
Supplement: Multimedia Appendix 1 [file medinform_v11i1e42736_app1.docx]

Appendix

**Diagnostic** **Uncertainty Algorithm Component Definitions:**

If yes to all three below, then diagnostic uncertainty exists:

1. Do there exist direct and/or indirect measures of diagnostic uncertainty within the initial admission evaluation (at least one of the below)?
   1. Direct measures include:
      1. Comment on questionable diagnosis with use of question marks, expressions of ‘unclear’ or unknown
      2. expansive differential diagnosis or differential with 2 or more ‘most likely’ diagnoses
      3. use of symptomatic descriptors in place of diagnosis
      4. discrepancy in assessment between different resident physician providers
   2. Indirect measures include:
      1. Initiation of additional diagnostic imaging, procedure or lab testing to differentiate between diagnoses
      2. consultation with additional providers in attempt to resolve unclear diagnosis
      3. provider implements risk averse disposition (i.e. observation, further monitoring for evolution of symptoms)
2. Did the admitting provider initially withhold definitive treatment for the ultimate diagnosis based on uncertainty or pending further evaluation at the time of admission?
3. After reading through documentation, do you believe the clinician experienced diagnostic uncertainty at the time of admission based on the operational definition, “a subjective perception experienced by the clinician that reflects their inability to provide an accurate explanation for the patient's health problem?”

Note: Our definition of diagnostic uncertainty includes direct and indirect measures of diagnostic uncertainty and an operational definition, which were adapted from Bhise and colleagues.^1^

**Diagnostic Uncertainty Algorithm:**

Do there exist direct and/or indirect measures of diagnostic uncertainty within the initial admission evaluation?

No

No diagnostic uncertainty

Did the admitting provider initially withhold definitive treatment for the ultimate diagnosis based on uncertainty or pending further evaluation at the time of admission?

Yes

After reading through documentation, do you believe the clinician experienced diagnostic uncertainty at the time of admission based on the operational definition, “a subjective perception experienced by the clinician that reflects their inability to provide an accurate explanation for the patient's health problem?”

Yes

Diagnostic uncertainty present

Yes

No

No diagnostic uncertainty

No

No diagnostic uncertainty

**Case Examples Using Diagnostic Uncertainty Algorithm:**

**Case** **1:** Uncomplicated Pneumonia, **Diagnostic Uncertainty Present**

History

- 11 year old female with history of asthma, allergic rhinitis, and recent flu-like illness with initial improvement, with one day of worsening cough and increased work of breathing
- Patient afebrile
- Placed on 2L oxygen at outside emergency room for desaturations
- WBC at outside hospital was within normal limits
- CXR from outside hospital with left lower lobe consolidation
- Treated at outside hospital with dose of ceftriaxone to cover for pneumonia and with albuterol and steroids for possible asthma exacerbation

Initial Admission Evaluation

- Highest on differential was atelectasis vs. viral pneumonia vs. viral pneumonitis vs. community acquired pneumonia
- Community acquired pneumonia thought to be less likely given afebrile, normal WBC and procalcitonin
- Decision made to monitor off antibiotics

Ultimate Diagnosis

- Community acquired pneumonia

**Diagnostic Uncertainty Algorithm:**

Do there exist direct and/or indirect measures of diagnostic uncertainty within the initial admission evaluation?

Yes, though only one is required, this case had presence of both a direct measure (differential with two or more ‘most likely’ diagnoses) and an indirect measure (additional labs to differentiate between diagnoses) of diagnostic uncertainty.

Did the admitting provider initially withhold definitive treatment for the ultimate diagnosis based on uncertainty or pending further evaluation at the time of admission?

Yes

After reading through documentation, do you believe the clinician experienced diagnostic uncertainty at the time of admission based on the operational definition, “a subjective perception experienced by the clinician that reflects their inability to provide an accurate explanation for the patient's health problem?”

Yes

Diagnostic uncertainty present

Yes

Explanation:

Yes, definitive treatment was withheld. Antibiotics for community acquired pneumonia were not ordered at the time of admission.

Yes, the reviewer believed the admitting clinician experienced diagnostic uncertainty at the time of admission based on the operational definition.

**Case 1 Example, Diagnostic Uncertainty Present**

No diagnostic uncertainty

No

No diagnostic uncertainty

No

Yes

Diagnostic uncertainty exists

Yes

After reading through documentation, do you believe the clinician experienced diagnostic uncertainty at the time of admission based on the operational definition, “a subjective perception experienced by the clinician that reflects their inability to provide an accurate explanation for the patient's health problem?”

**Case Examples Using Diagnostic Uncertainty Algorithm:**

**Case** **2:** Uncomplicated Pneumonia, **No Diagnostic Uncertainty**

History

- 8 year old previously healthy female presenting with eight days of fever, cough, congestion, headache.
- As an outpatient, had been diagnosed with sinusitis and started on amoxicillin. Later, diagnosed with clinical pneumonia outpatient and was transitioned between multiple different antibiotics.
- Noted to also have strawberry tongue, rash, conjunctivitis
- CXR with left lower lobe consolidation
- Respiratory viral panel was negative
- WBC, Hgb were normal. Platelets 171.
- ESR was elevated. CRP was normal.
- Albumin 2.5, AST and ALT mildly elevated.
- UA without pyuria

Initial Admission Evaluation

- Differential included community acquired pneumonia, incomplete Kawasaki, sinusitis
- Ultimately decided to proceed with treatment for pneumonia with ceftriaxone

Ultimate Diagnosis

- Community acquired pneumonia

Do there exist direct and/or indirect measures of diagnostic uncertainty within the initial admission evaluation?

Yes

Yes, a direct measure (differential with two or more ‘most likely’ diagnoses) was present.

Did the admitting provider initially withhold definitive treatment for the ultimate diagnosis based on uncertainty or pending further evaluation at the time of admission?

No

No diagnostic uncertainty

The admitting provider did NOT withhold definitive treatment for the ultimate diagnosis of community acquired pneumonia. Ceftriaxone was ordered to treat pneumonia at the time of admission.

Explanation:

**Diagnostic Uncertainty Algorithm:**

**Case 2 Example, No Diagnostic Uncertainty**

References

1. Bhise V, Rajan SS, Sittig DF, et al. Electronic health record reviews to measure diagnostic uncertainty in primary care. *Journal of Evaluation in Clinical Practice*. 2018;24(3):545-551. doi:10.1111/jep.12912
